# Acute Administration of Bioavailable Curcumin Alongside Ferrous Sulphate Supplements Does Not Impair Iron Absorption in Healthy Adults in a Randomised Trial

**DOI:** 10.3390/nu13072300

**Published:** 2021-07-03

**Authors:** Helena Tiekou Lorinczova, Gulshanara Begum, Derek Renshaw, Mohammed Gulrez Zariwala

**Affiliations:** 1 Centre for Nutraceuticals, School of Life Sciences, University of Westminster, 115 New Cavendish Street, London W1W 6UW, UK; w1505041@my.westminster.ac.uk (H.T.L.); begumru@westminster.ac.uk (G.B.); 2 Centre for Sport, Exercise and Life Sciences, Institute for Health and Wellbeing, Coventry University, Priory St, Coventry CV1 5FB, UK; derek.renshaw@coventry.ac.uk

**Keywords:** curcumin, iron, ferrous sulphate, supplementation, nutrition, antioxidant

## Abstract

Ferrous sulphate (FS) is a cost effective, readily available iron supplement for iron deficiency (ID). The pro-oxidant effect of oral ferrous iron is known to induce inflammation, causing gastric side-effects and resulting in poor compliance. Curcumin is a potent antioxidant and has also been shown to exhibit iron chelation in-vitro, although it is not established whether these effects are retained in-vivo. The aim of this study was therefore to assess the influence of a formulated bioavailable form of curcumin (HydroCurc^TM^; 500 mg) on acute iron absorption and status in a double blind, placebo-controlled randomized trial recruiting 155 healthy participants (79 males; 26.42 years ± 0.55 and 76 females; 25.82 years ± 0.54). Participants were randomly allocated to five different treatment groups: iron and curcumin placebo (FS0_Plac), low dose (18 mg) iron and curcumin placebo (FS18_Plac), low dose iron and curcumin (FS18_Curc), high dose (65 mg) iron and curcumin placebo (FS65_Plac), and high dose iron and curcumin (FS65_Curc). Participants were provided with the supplements according to their relevant treatment groups at baseline (0 min), and blood collection was carried out at 0 min and at 180 min following supplementation. In the treatment groups, significant difference was observed in mean serum iron between baseline (0 min) and at end-point (180 min) (F (1, 144) = 331.9, *p* < 0.0001) with statistically significant intra-group increases after 180 min (*p* < 0.0001) in the FS18_Plac (8.79 µmol/L), FS18_Curc (11.41 µmol/L), FS65_Plac (19.09 µmol/L), and FS65_Curc (16.39 µmol/L) groups. A significant difference was also observed between the two time points in serum TIBC levels and in whole blood haemoglobin (HGB) in the treatment groups, with a significant increase (1.55%/2.04 g/L) in HGB levels from baseline to end-point observed in the FS65_Curc group (*p* < 0.05). All groups receiving iron demonstrated an increase in transferrin saturation (TS%) in a dose-related manner, demonstrating that increases in serum iron are translated into increases in physiological iron transportation. This study demonstrates, for the first time, that regardless of ferrous dose, formulated curcumin in the form of HydroCurc™ does not negatively influence acute iron absorption in healthy humans.

## 1. Introduction

Iron is an essential mineral nutrient that plays a key role in most biological processes necessary to sustain life [1,2]. Iron deficiency (ID) is ranked as the most prevalent nutritional deficiency [3] affecting around 30% of the global population, equating to over 2 billion people [4,5]. ID is caused by inadequate dietary iron intake, as well as uptake by the body to replenish iron that is lost daily mainly due to erythrocyte turnover in addition to skin and gut cell sloughing. ID has been attributed to more than 60% of all anaemia cases worldwide and is associated with reduced life expectancy, altered immune response, developmental delays, and adverse pregnancy outcomes [3,6]. As iron is fundamental for growth and metabolism, ID even without anaemia can lead to impaired cognitive and physical development in children, compromise physical and cognitive performance in adults [3,7,8,9], and has been linked with fatigue [10], impaired quality of life [11], and reduced mood [3,9].

Ferrous sulphate is considered the global ‘gold standard’ iron supplement due to its extensive history of safe usage, documented effectiveness, and low cost [12,13]. However, non-compliance with ferrous sulphate is as high as 50% due to negative gastrointestinal (GI) effects attributed to iron intake [14,15,16]. The scope for ID treatment success is thus limited often by poor compliance, particularly due to adverse GI effects such as constipation, diarrohea, and flatulence [3]. These GI symptoms are often accompanied by increased inflammation as a consequence of excessive iron levels in the gut, facilitating the production of detrimental reactive oxygen species (ROS) and consequent damage to the gut mucosa [17].

Antioxidants can counteract the effects of ROS and have therefore generated interest as molecules that could potentially ameliorate the pro-oxidant side-effects of iron [18,19]. Curcumin, a non-flavonoid polyphenol, is the most biologically active antioxidant component in the rhizomatous spice *Curcuma longa* Linnaeus or turmeric [20]. Curcumin is comprised of three curcuminoids: curcumin (80–75%), demethoxycurcumin (DMC; 20%), and bisdemethoxycurcumin (BDMC; 2–5%) [21]. Curcumin has been extensively studied for its ROS scavenging properties [22,23,24,25]. It acts as a free-radical chain breaker, capable of donating hydrogen to ROS due to the presence of a hydroxyl group in its structure [26]. Curcumin also suppresses inflammation by mechanisms including downregulation of NF-κB (nuclear factor kappa-light-chain-enhancer of activated B cells), thereby demonstrating potent anti-inflammatory activity [27]. In addition to its antioxidant and anti-inflammatory action, curcumin has also been shown to have neuroprotective properties, particularly in relation to memory and cognition [28]. A meta-analysis of randomised controlled trials confirmed a significant positive impact of curcumin supplementation on levels of brain-derived neurotrophic factor (BDNF), a factor that affects cognitive function, learning, and memory [29]. Serum BDNF levels were also shown to increase significantly in healthy adults following co-administration of iron and curcumin supplements recently by Tiekou Lorinczova and co-workers [30]. The ability of curcumin to potentially counter iron-mediated ROS generation and inflammation in the GI tract, in concert with its ability to have a beneficial central effect, raises the intriguing possibility of co-administering iron and curcumin supplements to limit the oxidative stress associated with iron supplementation while synergising the beneficial effects of both molecules.

However, a body of research has shown whole turmeric or curcumin adversely affects iron absorption and iron status in animal models [31,32,33]. Whole turmeric is known to inhibit iron absorption by 20–90% in humans, reducing iron absorption in a dose-dependent manner [34]. Curcumin and its analogue curcuminoids, DMC and BDMC, can exist in two different forms depending on their environment, keto and enol [35]. In their enol form, they are capable of accepting as well as donating hydrogen and have metal chelation characteristics [36]. Curcumin binds ferric iron (Fe3+) to form a ferric–curcumin complex that is dose-dependent and Fe3+ specific [37]. 

Despite these observations, there remains considerable ambiguity relating to the nature of interactions between iron and curcumin in the context of absorption kinetics and physiological effects in humans. The aim of this research study was therefore to assess the influence of a formulated bioavailable form of curcumin (HydroCurc™) on acute iron absorption in humans. To the best knowledge of the authors, curcuminoid constituents have not been assessed previously in humans in relation to oral iron supplementation and in relation to circulatory iron levels.

## 2. Materials and Methods 

### 2.1. Study Design

Briefly, 155 healthy participants were recruited in a double blind, placebo-controlled randomized trial. Blocked, gender balanced randomisation was carried out using Study Randomizer software [38], and sample size was calculated using G*Power 3.1.9.2 software [39,40].

### 2.2. Ethics Approval and Study Registration

The study protocol was approved by the Faculty of Science and Technology Ethics Committee, University of Westminster under the application identification: ETH1718-0907. The study and the study protocol are registered with ClinicalTrials.gov (NCT04465851) and public since 7 June 2020.

### 2.3. Inclusion and Exclusion Criteria

All participants enrolled in the study aligned with the inclusion criteria of being healthy, aged 18–40 years, with ferritin levels in the normal range for the United Kingdom (UK; 15–300 µg/L for men and 15–200 µg/L for women) according to Dooley and Worwood, and Fitzsimons and colleagues [41,42]. Any prospective participants with deficient haemoglobin (HGB) levels (<130 g/L for men and <120 g/L for women) were not eligible for inclusion in the study [5].

Participants were excluded from the trial if they had any diagnosis of medical condition or comorbidities as per the exclusion criteria (such as, currently trying to conceive, pregnant, lactating, experiencing any chronic menstrual disorders, or reported undergoing any menopausal changes). Additionally, participants were excluded if they had any issues related to oral supplement ingestion; were on any medication or supplementation; if their alcohol consumption exceeded 21 units/week; or if they experienced any chronic GI symptoms, eating disorders, psychological conditions, or any hypo/hypertensive blood pressure (BP) measurements [43,44].

### 2.4. Safety Screening

All potential study participants were screened during a medical history interview for health conditions that may preclude participation in the study, conducted in 2018–2019, with respect to the inclusion/exclusion criteria. Interviews were supported with the use of the General Symptoms Questionnaire (GSQ-65). All participants were interviewed for a duration of 60 min and included the use of open-ended questions to gain comprehensive understanding of their health status. Furthermore, a fasting (12 h, overnight) blood sample was collected (for ferritin and full blood count analysis) and BP was measured using Omron M6 Comfort blood pressure monitor, (Omron, Hoofddorp, The Netherlands). Participants were not enrolled if anomalous results or findings from the safety screening were established.

### 2.5. Participants

**Figure 1:**
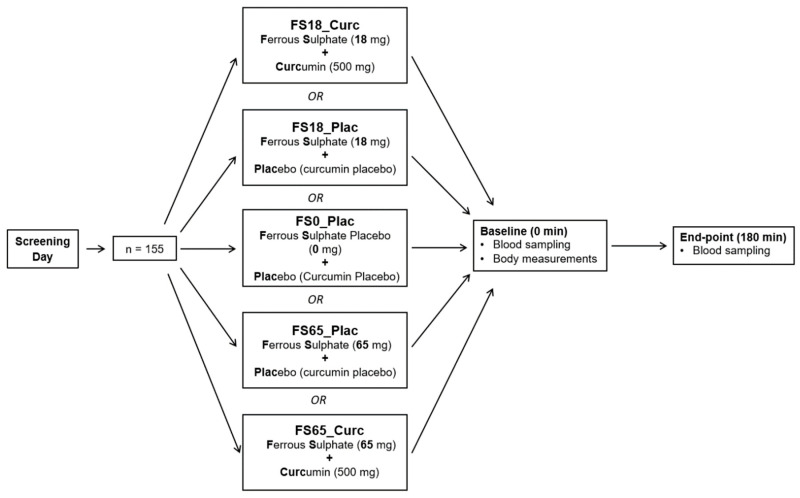
Study design. Random allocation to treatment groups post safety screening (*n* = 31/group). At baseline (0 min), both body measurements and blood samples were collected. At end-point (180 min), only blood samples were collected.

Study participants were randomly allocated to one of five different treatment groups using blocked, gender balanced randomisation (with 31 participants in each group) using the Study Randomizer software [38]. The five different treatment groups were ferrous sulphate placebo + curcumin placebo (FS0_Plac), ferrous sulphate–18 mg elemental iron + placebo (FS18_Plac), ferrous sulphate–18 mg elemental iron + 500 mg curcumin (FS18_Curc), ferrous sulphate–65 mg elemental iron + placebo (FS65_Plac), and ferrous sulphate–65 mg elemental iron +500 mg curcumin (FS65_Curc) (Figure 1).

### 2.6. Supplements

Participants were provided with two different doses of ferrous sulphate iron supplements depending on the group they were randomized to. The higher dose of ferrous sulphate was 200 mg with 65 mg elemental iron and is based upon the traditional first line oral iron therapy for treatment and prophylaxis of ID and ID anaemia generally used worldwide [45]. The lower dose was 55 mg ferrous sulphate containing 18 mg elemental iron, based upon the recommended Daily Value (DV) of iron as per the US Food and Drug Administration (FDA) [45,46]. Formulated curcumin supplement (HydroCurc™, Pharmako Biotechnologies Pty Ltd. New South Wales, Australia) included 500 mg/day comprising of 80% curcumin, 17% DMC, and 3% BDMC (~85% total curcuminoids), entrapped in a patented delivery system (LipiSperse^®^) [47]. For the placebo, microcrystalline cellulose was used. This cellulose was also used as an inert bulking agent in the capsules of active ingredients. The ferrous sulphate and curcumin supplements were packed into white-opaque hydroxypropyl methylcellulose (HPMC) capsules (sizes used were #1 and #00). Participants were provided with one ferrous sulphate iron at 0 min and with one curcumin supplement 60 min after the iron supplement on the day of the trial, in the fasted state, by trained research staff.

### 2.7. Physical Examination

Blood pressure was measured by a trained research associate or research assistant as described in the safety screening section. Anthropometric measurements were collected on the day of the study (baseline measures) by trained research staff. Height was measured to the nearest millimeter (without shoes) using a Seca 247 stadiometer (Seca GmbH & Co. KG, Hamburg, Germany). Weight, body mass index (BMI), and body fat % were measured using Seca mBCA 515 medical Body Composition Analyzer (Seca GmbH & Co. KG, Hamburg, Germany). Care was taken to instruct participants to void bladder of urine, empty pockets, and remove any excessive clothing and any jewellery prior to all measurements. Waist circumference was obtained using a soft tape measure at the narrowest point between the lower rib and the top of the hip bone. 

### 2.8. Blood Sample Collection and Processing

Blood tests were carried out for each participant, following an overnight fast (12-h fast). Venous blood samples were collected (by certified phlebotomists) by venipuncture (from the antecubital fossa) using a 21G needle, at time 0 min and 180 min after iron supplementation. Approximately 10 mL of blood was collected from each participant using a Becton Dickinson (BD) Vacutainer^®^ serum-separating tube (SST) and a BD Vacutainer^®^ ethylenediamine tetra-acetic acid (EDTA) tube (BD, Oxford, UK). Blood in the SSTs were left to coagulate at room temperature for 45 min and then centrifuged (Hettich 340r, Hettich GmbH & Co. KG, Tuttlingen, Germany). To render samples acellular, centrifugation was performed for 10 min at 5000 rpm/3857× *g* at 4 °C. Post centrifugation, aliquots of serum supernatant were stored at −80 °C in 1.5 mL microcentrifuge tubes. The EDTA tubes were kept on ice prior to whole blood analysis (on the day of collection) and then centrifuged for 10 min at 3857× *g* at 4 °C. Similarly, post centrifugation, aliquots of plasma supernatant were stored at −80 °C immediately.

### 2.9. Analysis of Serum Ferritin Concentration

Serum ferritin samples were defrosted and analysed using a Horiba ABX Pentra 400 (Horiba Ltd., Kyoto, Japan) multiparametric medical bench top chemistry analyser and the Horiba ABX Pentra CRP CP High Sensitivity and ABX Ferritin 2 CP reagents. This chemistry analyser is compliant with the National Committee for Clinical Laboratory Standards (NCCLS) [48]. In accordance with the manufactures processes, ferritin levels were determined by latex-enhanced immunoturbidimetric assay [49].

### 2.10. Analysis of Serum Iron Profile

Serum iron, total iron binding capacity (TIBC), transferrin saturation (TS), and unsaturated iron-binding capacity (UIBC) were assessed externally (using necessary standards and controls for each analyte) at Health Services laboratories (HSL Analytics LLP, London, UK); Project No. P197 as primary outcome measures of this study. Serum iron and UIBC were measured by direct colorimetric method, and subsequently, serum TIBC and TS were calculated using the equations from Elsayed et al. [50]:

TIBC = serum iron + UIBC

TS = serum iron/(serum iron +UIBC) × 100

### 2.11. Analysis of Whole Blood Haemoglobin (HGB) Concentration

HGB, a secondary outcome measure, was analysed using the Sysmex XP-300 (Sysmex Corporation, Kobe, Japan) automated haematology analyser. The Sysmex XP-300 is compliant with International Standards Organisation (ISO)/International Electrotechnical Commission (IEC) 17043:2010, via the Sysmex Network Communication Service. The Sysmex instrument aspirated 50 μL of each sample. HGB was analysed using a non-cyanide haemoglobin detection method [51]. The XP-300 was calibrated by Sysmex Corporation according to the manufacturer’s specifications, and quality was guaranteed using Sysmex internal quality control.

### 2.12. Analysis of Serum Thiobarbituric Acid Reactive Substances (TBARS) Concentration

Serum TBARS were determined by TBARS parameter assay kit (R&D Systems, Minneapolis, MN, USA), with a sensitivity of 0.007–0.055 μM minimum detectable dose range as another secondary outcome measure of the study. The assay procedure was performed according to the manufacturer’s instructions and plates were read using a microplate reader (SPECTROstar^®^ Nano, BMG Labtech GmbH, Ortenberg, Germany) at the absorbance of 530 nm.

### 2.13. Statistical Analysis

Values are expressed as mean ± Standard Error of Mean (SEM). All parameters were checked for normality using Shapiro–Wilk (S-W) tests, and results were statistically analysed using a two-way, repeated measures analysis of variance (ANOVA) or mixed effects model [52,53,54]. Post-hoc tests (Sidak’s and Tukey’s) were applied to assess differences between and within treatment groups with multiplicity adjusted P values reported for each comparison. Data analysis was carried out using PRISM software package (Version 9, Graphpad Software Inc., San Diego, CA, USA) and IBM Statistical Package for the Social Sciences (SPSS, Version 25.0, IBM Corp., New York, NY, USA). 

## 3. Results

### 3.1. Participant Characteristics

Of the 155 participants that started the study, there were 79 males (26.42 years ± 0.55) and 76 females (25.82 years ± 0.54). Participant 105 was excluded from further analysis (Figure S1) due to BMI of 49.65 kg/m^2^, the only BMI that fell in class 3 obese category (≥40 kg/m^2^) [55,56] and the only value detected as 3*IQR or an extreme outlier in SPSS. The age of participants ranged from 19 to 39 years of age. Overall, the mean age of the included 154 participants was 26.12 years (± 0.39). In addition, mean height was 1.71 m (±0.01), and mean weight was 68.93 kg (± 1.13). Anthropometric characteristics of participants within each of the five treatment groups are presented in Table 1.

### 3.2. Baseline Levels of Assessed Biomarkers

Baseline comparisons between each of the supplementation groups showed no statistical difference in any of the measured biological marker levels.

### 3.3. Effects of Acute Supplementation on Serum Iron Levels

**Figure 2:**
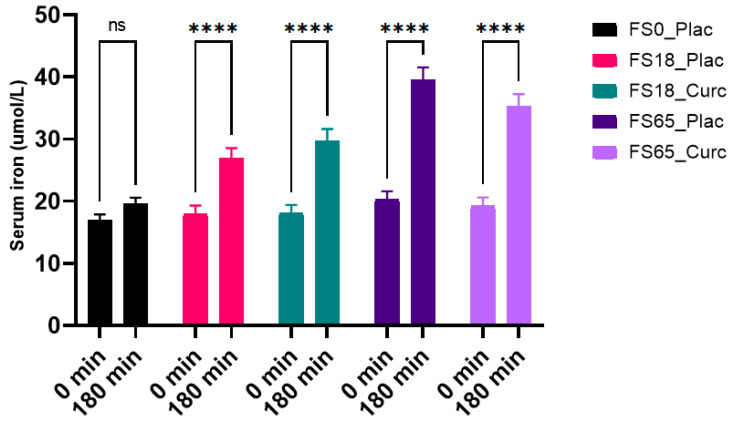
Serum iron levels at baseline and 180 min following supplementation. Results are presented as mean ± SEM Serum iron levels (µmol/L). ^ns^ indicates no significant difference (*p* > 0.05); * represents significance values when comparing time points within the same the conditions (**** *p* < 0.0001).

When assessing the effects of each treatment within the groups, there was a significant difference observed over time (F (1, 144) = 331.9, *p* < 0.0001) with statistically significant intra-group increases in mean serum iron after 180 min (*p* < 0.0001) in the FS18_Plac, FS18_Curc, FS65_Plac, and FS65_Curc groups. The lower dose groups, FS18_Curc and the corresponding curcumin placebo group FS18_Plac, after 180 min, had a 11.41 µmol/L and 8.79 µmol/L increase in serum iron, respectively. The higher dose groups, FS65_Curc and the corresponding iron and placebo group FS65_Plac, after 180 min, had 16.39 µmol/L and 19.09 µmol/L increase in serum iron, respectively. There was no significant increase observed in the FS0_Plac group (Figure 2).

Inter-group analysis of serum iron levels at 180 min showed significant between group difference (F (4, 149) = 10.73, *p* ≤ 0.0001). Statistically significant differences were observed between each treatment group except FS18_Plac vs. FS18_Curc, FS18_Curc vs. FS65_Curc and FS65_Plac vs. FS65_Curc (Table 2).

### 3.4. Effects of Acute Supplementation on Serum TIBC Levels

**Figure 3:**
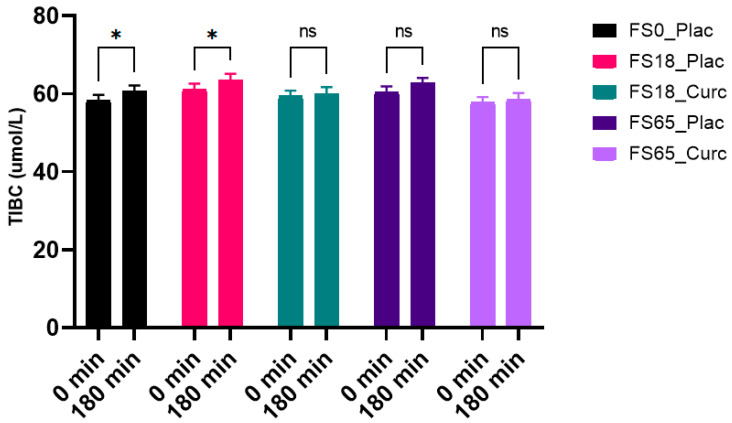
Effect of 180 min supplementation on mean serum total iron binding capacity (TIBC, µmol/L) levels (mean, SEM). * represents significance values when comparing time points within the same conditions (* *p* < 0.05); ^ns^ indicates no significant difference (*p* > 0.05).

When evaluating the effect of treatment group on serum TIBC levels after 180 min of supplementation, a significant difference was observed between the two time points (F (1, 137) = 19.76, *p* < 0.0001). A significant increase of 2.36 µmol/L (*p* = 0.0102) and 2.11 µmol/L (*p* = 0.0406) in TIBC levels from baseline to end-point was observed in the treatment groups FS0_Plac and FS18_Plac, respectively. There was no significant difference observed within the rest of treatment groups (FS18_Curc, FS65_Plac and FS65_Curc) over time (Figure 3). 

There were no significant differences observed in TIBC levels between groups 180 min after supplementation (F (4, 148) = 1.143, *p* = 0.3387).

### 3.5. Effects of Acute Supplementation on Serum UIBC Levels

**Figure 4:**
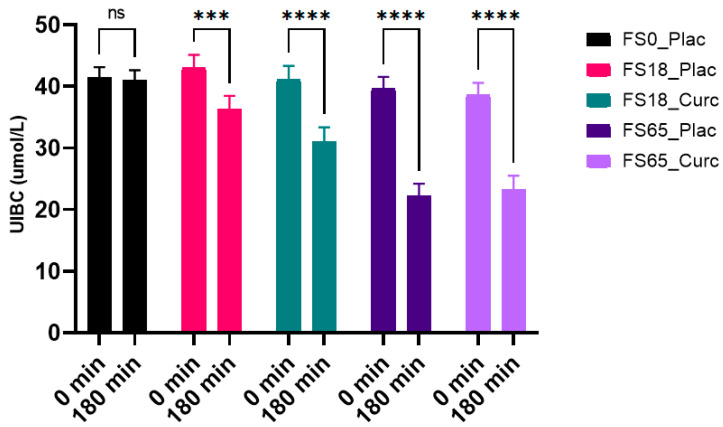
Effect of 180 min supplementation on mean serum unsaturated iron-binding capacity (UIBC, µmol/L) levels (mean, SEM). * represents significance values when comparing time points within the same the conditions (*** *p* = 0.0002, **** *p* < 0.0001); ^ns^ indicates no significant difference (*p* > 0.05).

When assessing the effects of each treatment within the groups, there was a significant difference observed over time (F (1, 137) = 210.8, *p* < 0.0001) with statistically significant intra-group decreases in mean serum UIBC levels in the FS18_Plac (6.61 µmol/L, *p* = 0.0002), FS18_Curc (9.69 µmol/L, *p* < 0.0001), FS65_Plac (18.06 µmol/L, *p* < 0.0001), and FS65_Curc (15.69 µmol/L, *p* < 0.0001) groups over time. There was no significant decrease observed in the FS0_Plac group (Figure 4).

When assessing the effects of each treatment on UIBC levels between groups at 180 min, a significant difference (F (4, 148) = 7.226, *p* < 0.0001) was detected. Statistically significant differences were observed between each treatment group except FS0_Plac vs. FS18_Curc, FS18_Plac vs. FS18_Curc, and FS65_Plac vs. FS65_Curc (Table 3).

### 3.6. Effects of Acute Supplementation on Serum Transferrin Saturation (TS) %

**Figure 5:**
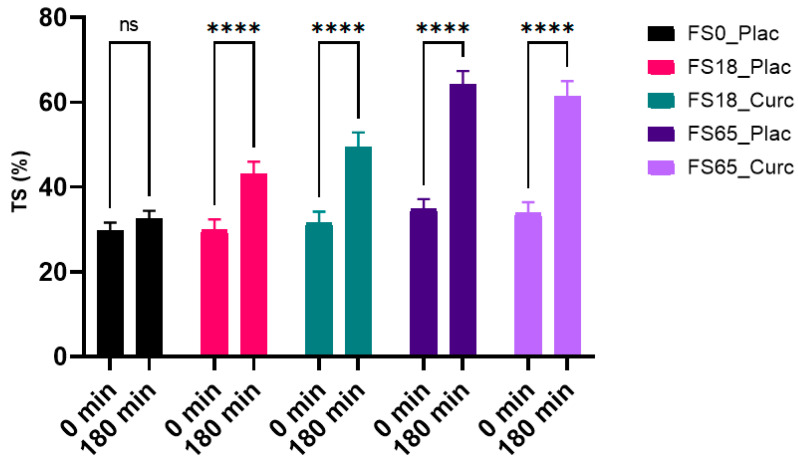
Effect of 180 min supplementation on mean serum transferrin saturation (TS) % (mean, SEM). * represents significance values when comparing time points within the same the conditions (**** *p* < 0.0001); ^ns^ represents no significant difference (*p* > 0.05).

When assessing the effects of each treatment on serum TS after 180 min of supplementation, a within group statistically significant difference was observed (F (1, 137) = 308.4, *p* < 0.0001). There were statistically significant (*p* < 0.0001) increases of 12.77%, 17.24%, 30.00%, and 27.96% in TS observed in the FS18_Plac, FS18_Curc, FS65_Plac, and FS65_Curc, respectively. There was no significant increase observed in the FS0_Plac group over time (Figure 5). 

When assessing the inter-group effects of each tretament on TS at 180 min, a significant difference (F (4, 148) = 10.39, *p* < 0.0001) was detected. Statistically significant differences were observed between each treatment groups except FS0_Plac vs. FS18_Curc, FS18_Plac vs. FS18_Curc, and FS65_Plac vs. FS65_Curc (Table 4).

### 3.7. Effects of Acute Supplementation on Whole Blood HGB Levels

**Figure 6:**
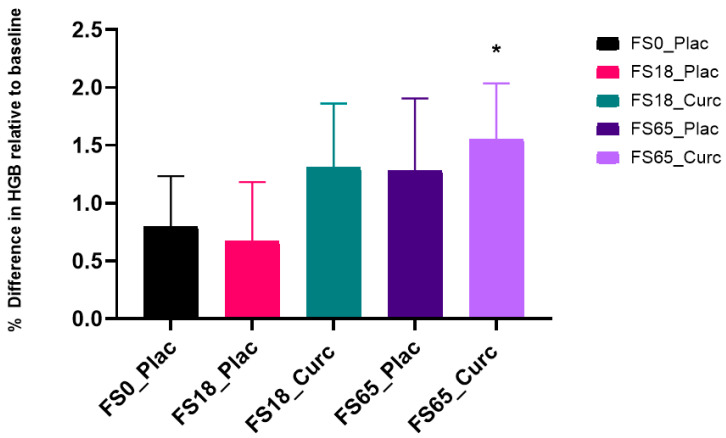
Haemoglobin (HGB) levels are expressed as percentage difference compared to baseline levels (SEM). * represents significant difference over time (* *p* < 0.05).

When evaluating the effect of treatment group on whole blood HGB levels after 180 min of supplementation, a significant difference was observed between the two time points (F (1, 147) = 22.35, *p* < 0.0001). A significant increase of 1.55%/2.04 g/L in HGB levels from baseline to end-point (180 min) was observed in participants taking FS65_Curc (* *p* < 0.05) (Figure 6 and Figure S2). 

There was no significant difference observed between treatment groups after 180 min of supplementation (F (4, 149) = 0.1042, *p* = 0.9809).

### 3.8. Effects of Acute Supplementation on Serum TBARS Levels

There were no significant intra- (F (1, 144) = 0.1889, *p* = 0.6645) or inter-group (F (4, 149) = 0.9583, *p* = 0.4323) differences observed in TBARS levels (level of oxidative stress) in this acute study as a result of iron and curcumin co-administration after 180 min.

## 4. Discussion

The polyphenol curcumin has been purported to have many health benefits as a result of its potential to target numerous signaling avenues and its impact on cellular level activity [57]. Despite its significant therapeutic potential, the pharmacological use of curcumin has been limited due to its poor bioavailability, limited bio-distribution, poor stability, and short activity half-life [58]. Formulation science strategies have shown that carrier-based delivery systems such as liposomes and micelles may address these limitations and enhance curcumin’s therapeutic potential [59,60,61]. This study utilised curcumin supplement in the form of HydroCurc™, a formulation consisting of 85% total curcuminoids entrapped in a proprietary delivery system (LipiSperse^®^) that has been shown to enhance bioavailability in humans [47].

Studies assessing the effects of curcumin on iron levels have provided mixed results. In murine models, it was shown that curcumin decreases systemic iron levels when administered consistently over several months [31,62,63]. Curcumin was shown to moderately chelate iron-forming insoluble complexes that may influence iron absorption in the gut, although this has largely been shown in in-vitro studies [64].

In the current study, co-administration of ferrous iron and curcumin in the form of HydroCurc™ does not negatively affect iron absorption compared to ferrous iron alone. Mean increases in serum iron after 180 min, in both FS65_Plac and FS65_Curc, are in line with expected increases in the literature [65,66,67,68]. Furthermore, HGB levels were also significantly elevated at 180 min compared to baseline in the group receiving the high iron dose of 65 mg ferrous sulphate with HydroCurc™. HGB increases can result from dehydration or a reduction in the plasma vascular fraction [69]. However, this is unlikely to explain the resulting increase in HGB levels as all participants across the study were given free access to drinking water ad libitum. 

Current understanding of iron homeostasis indicates that iron absorbed via the GI tract would first need to pass through the liver and bone marrow, before being incorporated into HGB during erythropoiesis [70]. However, this seems unlikely to explain the result in the current study given the short time frame of 180 min (3 h). Longitudinal research into iron supplementation, lasting several weeks (rather than hours), demonstrated significantly increased HGB levels in mildly anaemic populations [71]. However, the authors of the current study did not find any studies reporting acute changes (within 3 h) in HGB levels in a healthy population with normal serum levels. Although the researchers cannot rule out the possibility that this acute change is due to a physiological process, it cannot be explained based on current understanding of iron incorporation into HGB during erythropoiesis [70]. This outcome presents an intriguing avenue for in-depth exploration in further investigations.

The current study used serum iron analysis to evaluate iron absorption, as it increases in response to changes in dietary intakes of iron [3]. Dietary non-haem iron, typically found in the ferric (Fe^3^+) iron form, is oxidised into absorbable soluble ferrous (Fe^2^+) iron in the gastric and intestinal pH environment [72,73]. Given reports of diurnal changes in serum iron concentrations [74,75,76] and previous recommendations to standardize sampling [50], all participants’ baseline measures of serum iron were assessed in the morning following an overnight fast (12 h fast). Following randomization, all participants were observed to take oral ferrous sulphate on an empty stomach with water only (to circumvent any confounding effect of dietary components, which could influence iron absorption in an acute absorption test) [68]. To assess acute iron absorption from ferrous sulphate, it is advised to administer a dose yielding 65 mg of elemental iron and assess serum iron at 2–3 h post supplementation [67,68]. This method is used to evaluate gut iron absorption in humans [68,77,78]. If gut iron absorption is adequate, there is approximately a 17.9 µmol/L increase in serum iron levels as suggested by Alleyne et al. [68] or an increase of two to three times above baseline levels [67]. 

The current study also demonstrates that the increases in serum iron are translated into an increase in iron transportation via transferrin saturation levels. All groups receiving iron demonstrated an increase in transferrin saturation (TS%) and in a dose-related manner. Clinically, this measure is used as an indicator of iron-related diseases such as iron overload e.g., haemochromatosis or iron insufficiency e.g., iron deficiency anaemia [50]. However, in the current study, transferrin saturation (%) is an indicator of the saturation in iron transport capacity between different treatments and iron doses. Interestingly, there were no differences in transferrin saturation in the presence of HydroCurc™ compared to iron alone at either dose of iron. This finding indicates that HydroCurc™ has no effect on iron uptake or bioavailability at low or high doses of iron. Additionally, the dose response differences in transferrin saturation (%) as well as unsaturated iron binding capacity (UIBC) between the 18 mg and 65 mg groups indicates additional reserve capacity in the iron metabolism pathways in healthy participants to mobilise different doses of iron.

## 5. Conclusions

In the current study, regardless of ferrous dose (18 mg or 65 mg iron), formulated curcumin in the form of HydroCurc™ does not inhibit ferrous iron absorption following ingestion. Furthermore, the significant increase in HGB levels observed in the FS65_Plac group is retained in the FS65_Curc group, demonstrating that the serum iron levels result in a measurable physiological effect that is not diminished by the addition of formulated curcumin. To the best knowledge of the authors, this study has demonstrated for the first time that formulated curcumin does not appear to negatively influence acute iron absorption in healthy humans.

## Figures and Tables

**Table 1:** Anthropometric characteristics of participants split by gender.

Characteristics	FS0_Plac		FS18_Plac		FS18_Curc		FS65_Plac		FS65_Curc		Total	
	Male	Female	Male	Female	Male	Female	Male	Female	Male	Female	Male	Female
Age (years)	25.87 ± 1.18	26.73 ± 1.31	26.11 ± 1.27	25.08 ± 1.47	25.60 ± 1.13	23.53 ± 1.24	26.92 ± 1.25	27.06 ± 1.14	27.12 ± 1.35	26.36 ± 1.08	26.32 ± 0.55	25.81 ± 0.56
	(*n =* 15)	(*n =* 16)	(*n =* 18)	(*n =* 13)	(*n =* 15)	(*n =* 16)	(*n =* 13)	(*n =* 17)	(*n =* 17)	(*n =* 14)	(*n =* 78)	(*n =* 76)
Height (m)	1.77 ± 0.02	1.67 ± 0.02	1.76 ± 0.01	1.64 ± 0.01	1.77 ± 0.02	1.59 ± 0.02	1.78 ± 0.01	1.63 ± 0.02	1.78 ± 0.2	1.65 ± 0.02	1.77 ± 0.01	1.64 ± 0.01
	(*n =* 15)	(*n =* 15)	(*n =* 18)	(*n =* 12)	(*n =* 15)	(*n =* 15)	(*n =*13)	(*n =* 17)	(*n =* 17)	(*n =* 14)	(*n =* 78)	(*n =* 73)
Weight (kg)	75.22 ± 3.15	66.36 ± 3.25	80.70 ± 3.70	60.08 ± 3.07	74.58 ± 2.78	57.76 ± 2.64	77.13 ± 2.27	60.27 ± 2.89	75.24 ± 2.26	58.54 ± 1.91	76.69 ± 1.32	60.64 ± 1.28
	(*n =* 15)	(*n =* 15)	(*n =* 18)	(*n =* 12)	(*n =*15)	(*n =* 15)	(*n =* 13)	(*n =*17)	(*n =* 17)	(*n =* 14)	(*n =* 78)	(*n =* 73)
BMI (kg/m^2^)	24.06 ± 0.83	23.72 ± 0.85	25.94 ± 1.06	22.38 ± 1.21	23.71 ± 0.79	22.93 ± 1.03	24.32 ± 0.56	22.46 ± 0.82	23.95 ± 0.77	21.48 ± 0.65	24.44 ± 0.38	22.61 ± 0.41
	(*n =* 15)	(*n =* 15)	(*n =* 18)	(*n =* 12)	(*n =* 15)	(*n =* 15)	(*n =* 13)	(*n =* 17)	(*n =*17)	(*n =*14)	(*n =* 78)	(*n =* 73)
Body Fat (%)	18.37 ± 1.68	32.16 ± 1.57	21.97 ± 2.02	29.45 ± 2.44	18.35 ± 1.86	30.29 ± 2.07	19.13 ± 1.51	27.95 ± 1.52	19.44 ± 1.85	28.18 ± 1.25	19.56 ± 0.82	29.59 ± 0.79
	(*n =* 15)	(*n =* 15)	(*n =* 18)	(*n =* 12)	(*n =* 15)	(*n =*15)	(*n =* 13)	(*n =* 17)	(*n =* 17)	(*n =* 14)	(*n =* 78)	(*n =* 73)

Values presented in mean ± standard error of mean (SEM).

**Table 2:** Inter-group differences in serum iron levels (µmol/L) at 180 min.

Groups		MD ± SED		Sig. *	*p−*Value
FS0_Plac	vs.	FS18_Plac	−7.18 ± 2.18	**	0.0096
FS0_Plac	vs.	FS18_Curc	−10.02 ± 2.16	****	<0.0001
FS0_Plac	vs.	FS65_Plac	−19.88 ± 2.17	****	< 0.0001
FS0_Plac	vs.	FS65_Curc	−15.63 ± 2.16	****	<0.0001
FS18_Plac	vs.	FS18_Curc	−2.84 ± 2.19	NS	0.6917
FS18_Plac	vs.	FS65_Plac	−12.71 ± 2.19	****	<0.0001
FS18_Plac	vs.	FS65_Curc	−8.45 ± 2.19	**	0.0013
FS18_Curc	vs.	FS65_Plac	−9.86 ± 2.18	****	<0.0001
FS18_Curc	vs.	FS65_Curc	−5.61 ± 2.18	NS	0.0771
FS65_Plac	vs.	FS65_Curc	4.25 ± 2.18	NS	0.2935

Results presented as mean difference (MD) ± standard error of difference (SED). Sig.* represents significance values when comparing groups at the same timepoint (** *p* < 0.01, **** *p* < 0.0001, NS *p* > 0.05).

**Table 3:** Inter-group differences in serum unsaturated iron-binding capacity (UIBC) levels (µmol/L) at 180 min.

Groups		MD ± SED		Sig. *	*p-*Value
FS0_Plac	vs.	FS18_Plac	4.57 ± 2.80	NS	0.4782
FS0_Plac	vs.	FS18_Curc	9.56 ± 2.78	**	0.0062
FS0_Plac	vs.	FS65_Plac	19.43 ± 2.83	****	<0.0001
FS0_Plac	vs.	FS65_Curc	18.10 ± 2.81	****	<0.0001
FS18_Plac	vs.	FS18_Curc	4.99 ± 2.83	NS	0.3944
FS18_Plac	vs.	FS65_Plac	7.00 ± 2.86	****	<0.0001
FS18_Plac	vs.	FS65_Curc	13.53 ± 2.85	****	<0.0001
FS18_Curc	vs.	FS65_Plac	9.87 ± 2.85	**	0.0055
FS18_Curc	vs.	FS65_Curc	8.54 ± 2.84	*	0.0235
FS65_Plac	vs.	FS65_Curc	−1.33 ± 2.87	NS	0.9906

Results presented as mean difference (MD) ± standard error of difference (SED). Sig.* represents significance values when comparing groups at the same timepoint (* *p* < 0.05, ** *p* < 0.01, **** *p* < 0.0001, NS *p* > 0.05).

**Table 4:** Inter-group differences in transferrin saturation (TS %) at 180 min.

Groups		MD ± SED		Sig. *	*p−*Value
FS0_Plac	vs.	FS18_Plac	−10.06 ± 3.78	NS	0.0627
FS0_Plac	vs.	FS18_Curc	−16.30 ± 3.76	***	0.0002
FS0_Plac	vs.	FS65_Plac	−32.20 ± 3.82	****	<0.0001
FS0_Plac	vs.	FS65_Curc	−29.21± 3.80	****	<0.0001
FS18_Plac	vs.	FS18_Curc	−6.24 ± 3.82	NS	0.4772
FS18_Plac	vs.	FS65_Plac	−22.14 ± 3.88	****	<0.0001
FS18_Plac	vs.	FS65_Curc	−19.14 ± 3.85	****	<0.0001
FS18_Curc	vs.	FS65_Plac	−15.90 ± 3.86	***	0.0005
FS18_Curc	vs.	FS65_Curc	−12.90 ± 3.83	**	0.0077
FS65_Plac	vs.	FS65_Curc	2.99 ± 3.89	NS	0.9388

Results presented as mean difference (MD) ± standard error of difference (SED). Sig.* represents significance values when comparing groups at the same timepoint (** *p* < 0.01, *** *p* < 0.001, **** *p* < 0.0001, NS *p* > 0.05).

## Data Availability

The data presented in this study are available on request from the corresponding author. The data are not publicly available due to ethical, legal and privacy issues.

## Supplementary Materials

The following are available online at https://www.mdpi.com/article/10.3390/nu13072300/s1, Figure S1: The study compliance after 155 participants were enrolled and randomised equally into 5 treatment groups; Figure S2: Effect of 180 min supplementation on mean Haemoglobin (HGB) levels.
