# Supplementary material for: Acute Administration of Bioavailable Curcumin Alongside Ferrous Sulphate Supplements Does Not Impair Iron Absorption in Healthy Adults in a Randomised Trial
Source: Nutrients. 2021 Jul 3;13(7):2300. doi: 10.3390/nu13072300 (PMC8308323; doi:10.3390/nu13072300)
Supplement: Supplementary file 1 [file nutrients-13-02300-s001.zip › nutrients-1258863-supplementary.pdf]

Supplementary figures (Figure S1 and Figure S2) for the manuscript '**Acute administration of bioavailable curcumin alongside ferrous sulphate supplements does not impair iron absorption in healthy adults in a randomised trial'**

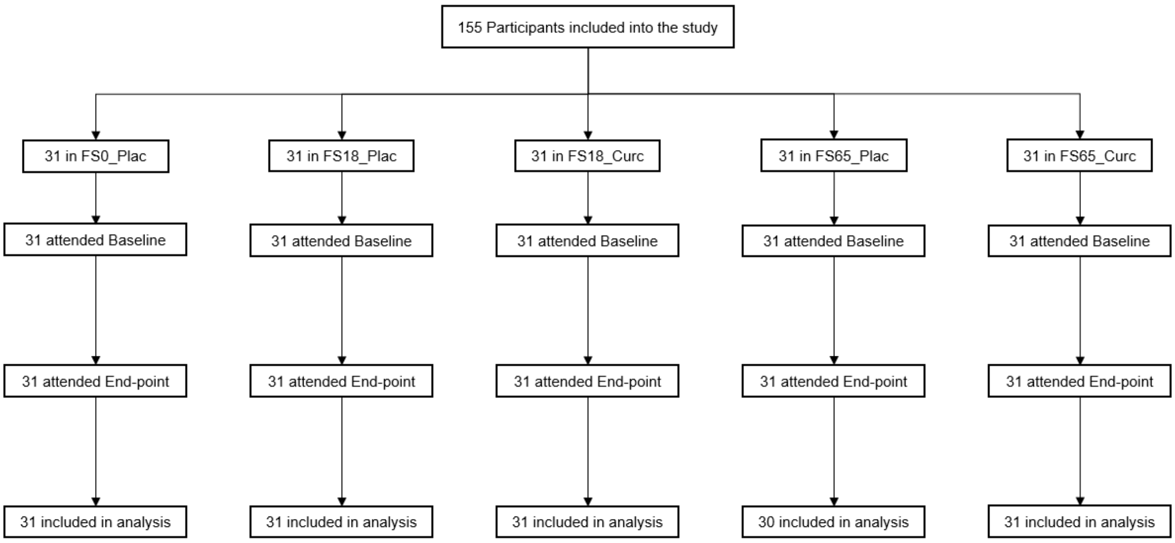

Figure S1: Study compliance of the 155 participants enrolled and equally randomised into 5 treatment groups: FS0_Plac (full placebo, placebos for both iron and curcumin), FS18_Plac (18 mg elemental iron and placebo for curcumin), FS18_Curc (18 mg elemental iron and 500 mg curcumin), FS65_Plac (65 mg elemental iron and placebo for curcumin) and FS65_Curc (65 mg elemental iron and 500 mg curcumin). From the 155 participants who completed the study 154 were enrolled in the data analysis.

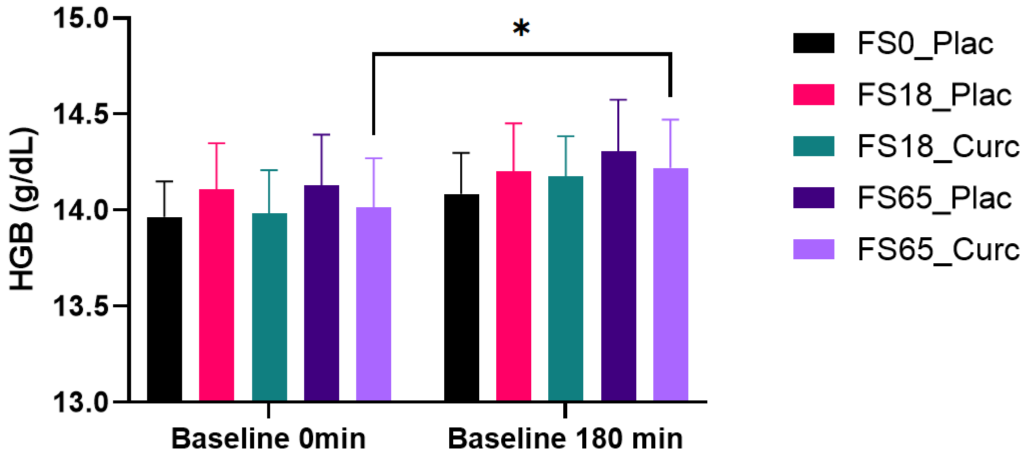

Figure S2. Effect of 180 min supplementation on mean Haemoglobin (HGB) g/dL (mean, SEM). *represents significant difference over time (* $p < 0.05$).
